# Experimentation and Predictive Models for Properties of Concrete Added with Active and Inactive SiO_2_ Fillers

**DOI:** 10.3390/ma12020299

**Published:** 2019-01-18

**Authors:** Wasim Abbass, Mohammad Iqbal Khan, Shehab Mourad

**Affiliations:** Department of Civil Engineering, King Saud University, Riyadh 800-11421, Saudi Arabia; wabbass@ksu.edu.sa (W.A.); smourad@ksu.edu.sa (S.M.)

**Keywords:** ultrafines, silica fume, mechanical properties, chloride ion penetrability, predictive models

## Abstract

Cement is one of the main constituents of concrete material and it is one of the main sources of carbon dioxide emissions in the environment. Fillers within a range of 5–7% from different sources can be used as a replacement of cement without compromising the properties of concrete or even tailoring for required property. This paper investigates the influence of inactive silica filler and silica fume on the mechanical- and durability-related properties of concrete with different strengths. The investigated mechanical properties focused on compressive strength at different ages up to 400 days, while the durability-related properties focused on porosity and rapid chloride ion penetrability (RCPT). Two types of ultrafines, namely quartz ultrafine and silica fume, were used. Concrete mixtures with four different water/binder ratios (0.25, 0.30, 0.35, 0.40) were prepared for various dosages of quartz ultrafine (0%, 5%, 8%, 10%, 15%, 25%, and 35%) and different dosages of silica fume (0%, 8%, 10%, and 12%). The results revealed that the compressive strength and durability related properties of concrete with different dosages of ultrafines and silica fume were significantly affected; however, there was a negative impact of ultrafine filler on the compressive strength after replacement of more than 8% of ultrafines. The strength relationships for the concrete with different water-to-cement ratio were assessed and certain modifications were proposed for ultrafines and silica fume. Predictive models were proposed for predicting the compressive strength of concrete in terms of RCPT and porosity for different levels of replacements of ultrafines and silica fume.

## 1. Introduction

Concrete is a heterogeneous material composed itself of different materials such as coarse aggregate, fine aggregate, cement, etc. The physical and chemical properties of these materials significantly affect the properties of concrete. One of the most important parts of such materials are fine fillers which compensate for the irregularities of aggregates available in concrete [1]. Fine fillers can be a better alternate for the replacement of cement, and consequently, can aid in reducing emissions of carbon dioxide in atmosphere. Almost one ton of carbon dioxide is generated through the production of one ton of cement, i.e., clinker fabrication releases around 825 kg CO_2_ per ton of clinker [2]. Consequently, higher levels of replacement of cement will reduce emission of CO_2_ in the environment. Concrete with higher mechanical and durability properties are prepared through dense structure of matrix. The densification of matrix is achieved by addition of ultrafine minerals and chemical actions. Physical densifying through properly grading material in the matrix will also prepare more compacted structures [3]. The chemical action (pozzolanic reaction) is explained by fixing of lime release during cement hydration to form a new generation of more compact C–S–H, which improves the performance and long-term durability of concrete [4,5,6]. Consequently, the use of mineral admixture which is chemically active or inert in the concrete material has increased in recent years due its environmental benefits (reduced cement consumption) and technical advantages (improved mechanical and durability related properties). Silica fume is one of the major mineral admixtures which has been widely used in construction since the 1970s and which is still the focus of research due to its physical and chemical properties [7,8,9,10,11].

In the last few years, the use of mineral admixtures which are either inert or having very low reactivity in cement-based material, along with very fine sizes, has captured the attention of researchers due to the increase in demand of new mineral additives. In addition, researchers have focused on utilizing fine by-products such as blast furnace slag and fly ash for use as fine fillers for concrete, especially when they are already available in some regions. Utilizing such fine fillers in concrete has reported positive effects. As one example, grinding of sugarcane bagasse ash with high levels of quartz with reduction in its particle size has caused increase in pozzolanic activity [12,13]. In addition, increase in the degree of hydration of mortar with inclusion of quartz particles (10–75%) was observed with respect to reference mortar [14,15]. Moreover, reduced particle size of mineral admixtures affects hydration along with improving nucleation sites; these nucleation sites enhance with the reduction in particle size of mineral admixtures [16]. 

It was reported that ultrafine filler improves the strength properties of concrete by using quartz filler and higher water-to-cement ratios [17]. The results of adding ultrafines to concrete has different effects on its strength properties based on the particle size of the ultrafines. Quartz ultrafines with a size less than 5 µm needs further investigation to understand its effect on the hardened properties of concrete. Until now, researchers have used quartz ultrafine as inert filler for strength, but how it affects the strength properties with time and permeation-related properties still needs to be explored in comparison with high pozzolanic active silica, i.e., silica fume. 

In this research work, the use of ultrafine quartz and silica as a partial replacement of cement in concrete mixtures with different water-to-cementitious ratios were examined extensively to investigate its effect on the compressive strength at different ages and durability related properties. The durability-related properties focused on were chloride ion penetrability, porosity, and rapid chloride ion penetrability (RCPT). Wider ranges of ultrafine ratios were used to find out the optimal content of ultrafine quartz and silica fume as partial replacements of cement with respect to the strength and durability related properties of concrete. Two types of ultrafines, namely quartz ultrafine and silica fume, were used. Concrete mixtures with four different water/binder ratios (0.25, 0.30, 0.35, 0.40) were prepared for various dosages of quartz ultrafine (0%, 5%, 8%, 10%, 15%, 25%, and 35%) and different dosages of silica fume (0%, 8%, 10%, and 12%). 

## 2. Materials 

### 2.1. Cementitious Materials 

**Figure 1:**
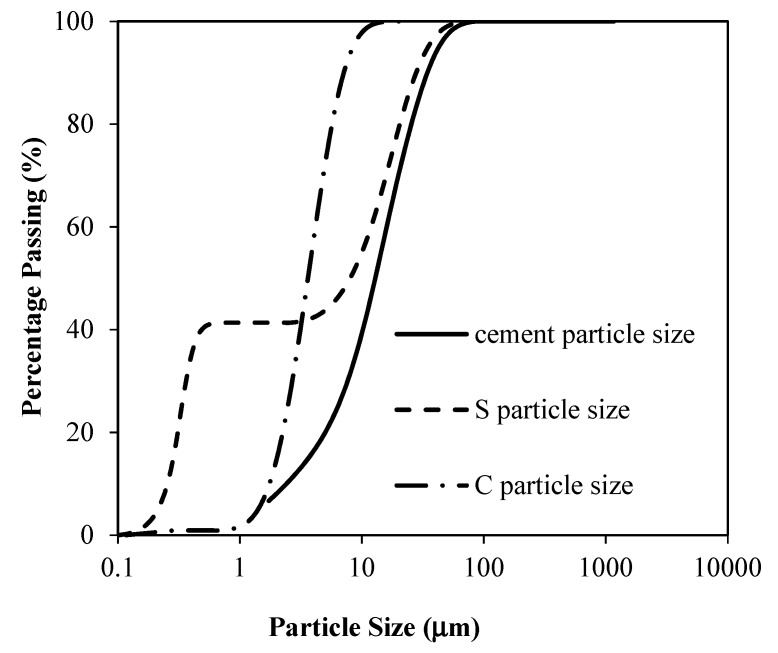
Particle size distribution of cement, silica fume, and quartz filler.

**Figure 2:**
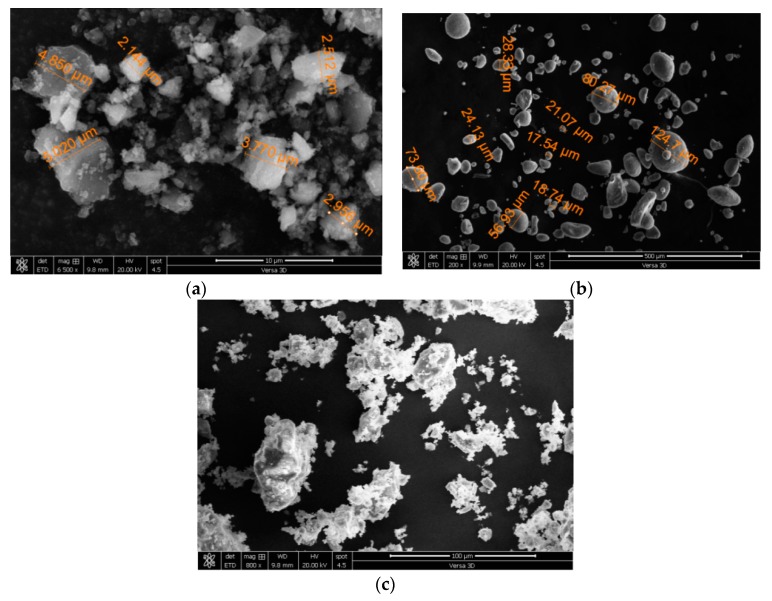
Scanning electron microscopy (SEM) images of fine materials. (**a**) Ultrafine (C); (**b**) Silica fume (S); (**c**) Cement.

Ordinary Portland cement (OPC) according to ASTM C150 [18] requirements was used as binder material which was partly replaced with silica fume. Median grain sizes of OPC, silica fume, and quartz ultrafine were 13 µm, 8 µm, and 3.5 µm, respectively. The physical and chemical properties of the abovementioned fine materials are shown in Table 1. The particle size distributions of the fine materials are shown in Figure 1. Silica fume consists of very fine particles with particle sizes of less than 1 µm. Particle size distribution of silica fume showed that around 40% of particles are less than 1 µm and about 60% of particles are between 6 and 10 µm, whereas use of well-graded silica fume has a particle size of 0.1 µm [3]. This type of grading makes it more interesting to study its effect on hardened and durability related properties of a material. The scanning electronic microscopic (SEM) image of fine material is shown in Figure 2. The SEM images of silica fume and ultrafines shows the particles with a size of less than 10 µm. The ultrafine filler shows irregular particles, whereas silica fume particles have a quite spherical-like shape, as shown in Figure 2.

### 2.2. Fine and Coarse Aggregates

**Figure 3:**
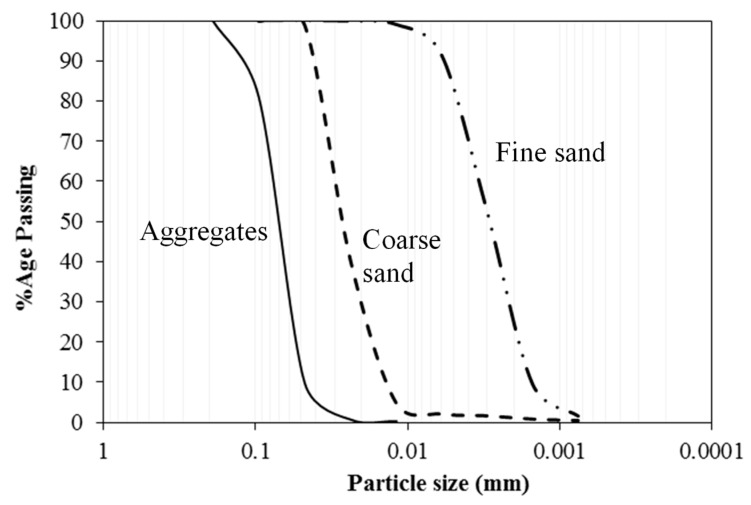
Particle size distribution of fine and coarse aggregate.

Two types of sands with different particle sizes were used for preparation of mixes named as crushed sand and fine sand. The Fineness moduli were 1.47 and 4.66 for fine and crushed sand, respectively. Fine and crushed siliceous sand were combined as 65% and 35%, respectively, to achieve a fineness modulus of 2.54. Coarse aggregate with maximum size of 10 mm was used for mix preparation. The physical properties of fine and coarse aggregate are summarized in Table 2. Particle size distributions of sands and coarse aggregate are shown in Figure 3.

### 2.3. Chemical Admixtures

A modified polycarboxylic ether (PE) polymer (Glenium 51) was used in the production of required mixture. It had a specific gravity of 1.1 and dry extract of 36%. The dosage of PE is expressed as a dry extract (DE) per cement weight. The PE dosage was optimized for workable mixture of concrete. 

## 3. Mix Preparations and Test Setup

### 3.1. Mix Properties and Preparations

The experimental work includes the preparation of eight different concrete mixes with four different water/binder ratios (0.25, 0.30, 0.35, 0.40) with various dosages of quartz ultrafine (0%, 5%, 8%, 10%, 15%, 25%, and 35%) and different dosages of silica fume (0%, 8%, 10%, and 12%). Table 3 shows the mix proportions of different mixes. In the mix design, cement content of 550 kg/m^3^ for 0.25 w/c ratio, 500 kg/m^3^ for 0.30 w/c ratio, 450 kg/m^3^ for 0.35 w/c ratio, and 400 kg/m^3^ for 0.40 w/c ratio were chosen. The amount of coarse aggregate used for the mixtures was 1056 kg/m^3^. The slump of all mixtures was attempted to be maintained between 175 ± 25 mm as shown in Table 3.

All aggregates were added to the mixer and mixed for a few revolutions with absorption water and then the fines were added and mixed in dry state for few minutes. Premixed super plasticizer in water was added to aggregates and mixed for three minutes followed by a rest for three minutes, then mixed again for two minutes. The mixer was stopped followed by casting of the specimen in molds. Concrete cylinders of 100 × 200 mm were casted for standard compressive strength tests in rigid plastic molds, while concrete discs 100 mm in diameter and 50-mm thick were casted in molds for durability according to ASTM 1202 [19] and porosity tests.

### 3.2. Experimental Method

The compressive strength and modulus of elasticity of high-strength concrete was measured with a 100 × 200 cylinder at a loading rate of 0.25 MPa/s. A water saturated, 50-mm thick and 100-mm diameter concrete sample was subjected to 60 V applied DC voltage for 6 h. Concrete saturated samples were placed in two reservoirs. One reservoir of 3% NaCl and another reservoir of 0.3 M NaOH solution covered the saturated concrete sample for the entire duration of the test. The charge passed was noted and the concrete was characterized according to the table given in ASTM 1202 [19]. 

The porosity test was done by drying the concrete disc samples (100 mm in diameter and 50-mm thick) at 100 ± 5 °C until constant weight was achieved. After drying the samples to constant weight, they were placed in desiccator under vacuum for three hours followed by immersion in the de-aired distilled water for one hour to attain saturated sample of concrete. Porosity of concrete was measured by Equation (1):

(1) $$P=\frac{W_{ss}-W_{d}}{W_{ss}-W_{w}}\times 100$$

where $W_{ss}$ is the weight of the saturated sample in air, $W_{d}$ is the weight of oven-dried sample, $W_{w}$ is the weight of sample in water, and *P* is the porosity of concrete in percentage. 

## 4. Results and Discussion

### 4.1. Effect of Cement Replacement on the Properties of Concrete at 28 Days

**Figure 4:**
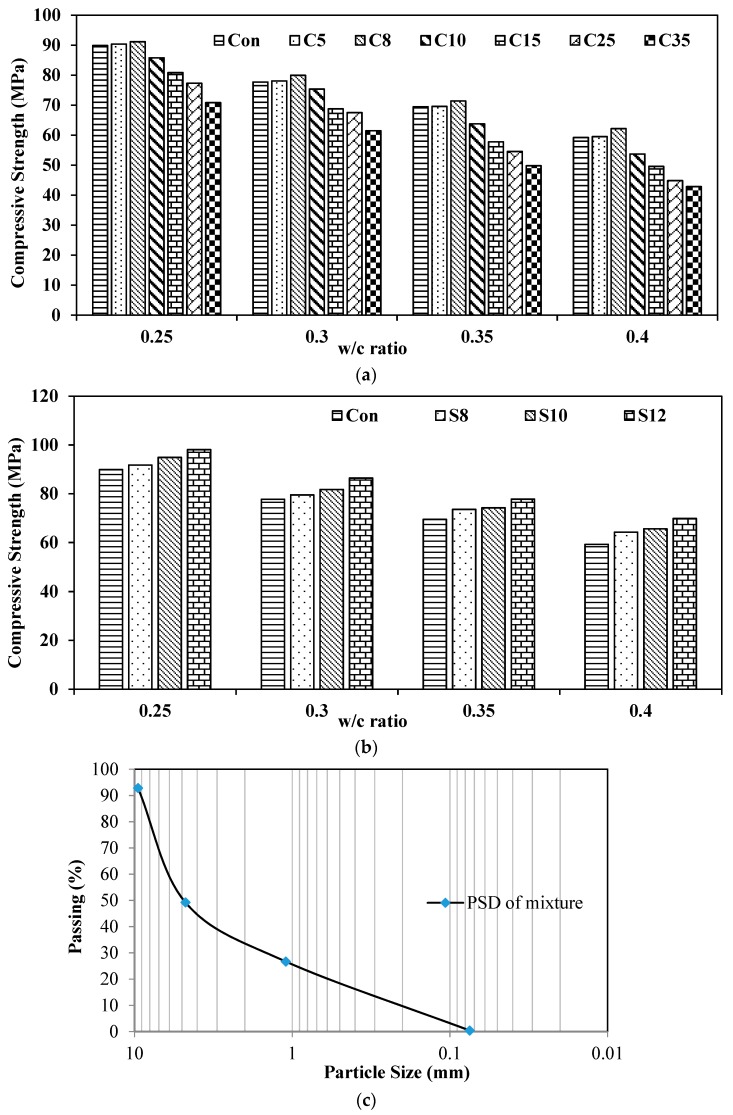
Compressive strength vs. w/c ratio for ultrafine and silica fume dosages. (**a**) Compressive strength vs. w/c ratio for UF dosages; (**b**) compressive strength vs. w/c ratio for SF dosages; (**c**) particle size distribution of concrete mixtures.

The effect of ultrafines and silica fume on the compressive strength for different water to cementitious ratios ranging from 0.25, 0.3, 0.35, 0.4 are shown in Figure 4. Figure 4a shows the results of different dosages of ultrafines in different water-to-cement ratios. For all water-to-cement ratios, no reduction was observed in the compressive strength of the matrix with dosages less than 10% of ultrafine. There was a slight increase in the compressive strength with the replacement of 8% of cement with ultrafine. However, once the dosage of ultrafine was increased from 10%, a decrease in compressive strength was observed at the age of 28 days. Such a decrease in compressive strength was more pronounced in higher water-to-cement ratios. The maximum reduction in compressive strength for 15%, 25%, and 35% replacement of ultrafine was observed to be 16%, 24%, and 28% respectively at the age of 28 days for w/c ratio of 0.4. Such a reduction in strength may be attributed due to the availability of lesser pozzolanic reaction of ultrafines. The results show that there is a slight increase with lower dosage until 8% replacement of ultrafines, which may be attributed due to the fact that optimum packing of paste at the micro level is in accordance with other studies [20,21,22,23]. Silica fume is a very fine material and normally used as a property enhancing material. The effect of three different dosages of silica fume with four different water-to-cement ratios is shown in Figure 4b at the age of 28 days. The hardened properties of concrete can directly be related to chemical and physical mechanisms through which silica fume functions. The physical mechanism by which silica fume affects concrete consists of reduction in bleeding, facilitation of nucleation site, and enhanced particle packing of solids. Change of pore structure, paste aggregate transition zone, and chemical compositions are incorporated due to the addition of silica fume in concrete. More of the enhancement in mechanical properties of concrete are due to increased bond strength of paste and aggregate [24]. The increase in the strength of concrete is reported by many studies [25,26,27] to be 30 to 100 percent depending upon the mix type, dosage of silica fume, use of superplasticizer, type of aggregate, cement type, and curing regime. The major physical effect of silica fume in concrete is improvement in packing of particle because finer silica fume particles can fill the spaces in between cement grains just as sand fills the gap between coarse aggregate particles. The particle size distribution of concrete mixture is shown in Figure 4c. This filling of spaces between cement grain particles is valid only once sufficient amount of super plasticizer is available to neutralize the attractive surface forces [28]. Figure 4b is showing that the addition of three dosages of silica fume has increased the compressive strength of concrete with four different water-to-cement ratios 0.25, 0.3, 0.35, 0.4 at the age of 28 days. The increase in compressive strength for 8%, 10%, and 12% replacement at water-to-cement ratio of 0.25 is 2%, 5%, and 9%, respectively. At the water-to-cement ratio of 0.3 and the replacement of silica fume with dosages of 8%, 10%, and 12%, the increases in compressive strength were 3%, 5%, and 11%, respectively. At the water-to-cement ratio of 0.35 and the replacement of silica fume with dosages of 8%, 10%, and 12%, the increases in compressive strength were 6%, 7%, and 12%, respectively. At the water-to-cement ratio of 0.4 and the replacement of silica fume with dosages of 8%, 10%, and 12%, the increases in compressive strength were 8.5%, 11%, and 18%, respectively. As it can be observed from the abovementioned results, a maximum increase in the compressive strength of concrete of 18% was observed for the water-to-cement ratio of 0.4 at a replacement level of 12% of silica fume. It can be observed that as the water-to-cement ratio of matrix increased, the addition of silica fume was pronounced in the enhancement of compressive strength, which may be attributed because at higher water-to-cement ratios, the more porosity between the cement grains is available. These micro-gaps between cement particle were supposed to be filled by the silica fume particles, and hence, the compressive strength of the material enhanced. At the lower water-to-cement ratios of 0.25 and 0.30, the matrix structure was already been well compacted while achieving the workability of material by the addition of superplasticizer, but still these finer particles were enhancing the strength properties of concrete as shown in Figure 4b.

### 4.2. Age Effect on the Compressive Strength of Concrete

**Figure 5:**
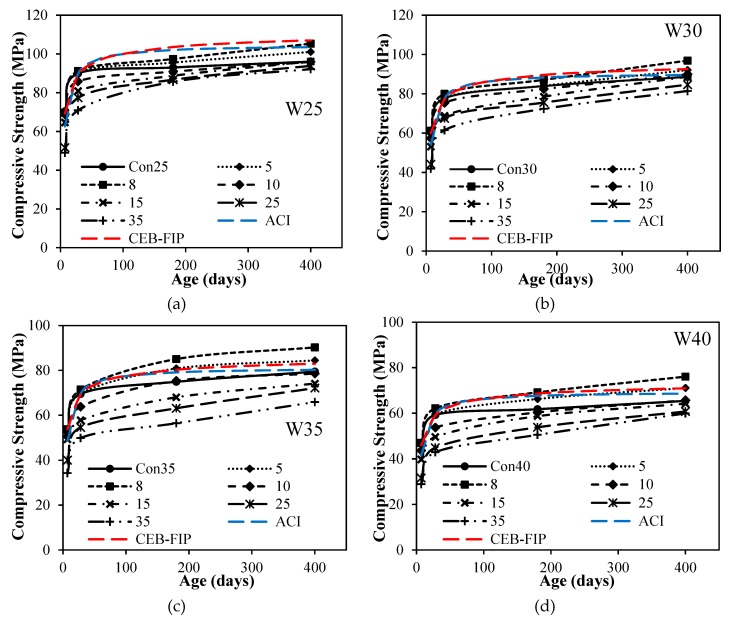
Compressive strength vs. age for different ultrafine dosages with different w/c ratios: (**a**) compressive strength vs. age for different ultrafine dosages at w/c 0.25; (**b**) compressive strength vs. age for different ultrafine dosages at w/c 0.30; (**c**) compressive strength vs. age for different ultrafine dosages at w/c 0.35; (**d**) compressive strength vs. age for different ultrafine dosages at w/c 0.40.

There are different parameters that affect the rate of gain of strength with time. Among those parameters, the type and strength class of cement, amount and type of mineral admixture and additions, water-to-binder ratio and curing conditions. The relationship presented for the time–strength has assumptions of moist curing and normal temperature. For a constant water-to-cement ratio, the higher strength was predicted for longer exposure of moist curing condition, considering that the anhydrous cement particles were going on under hydration. There was no increase in strength with time observed, as the water was lost through the capillaries due to air-curing conditions [29]. There is no significant increase or minimum increase in compressive strength with addition of silica fume after the age of 28 days [24]. There is very limited literature available which has reported the apparent reduction in compressive strength due to the addition of silica fume in concrete at an age of more than 90 days [30,31,32,33]. The ACI 234 reported that reduction in compressive strength may be due to testing machines or other factors which affect strength [24]. The compressive strength development for concrete with ultrafine and with different water-to-cement ratios are presented in Figure 5. Two empirical equations provided by the ACI Committee 209 [34] and CEB-FIP Models Code [35] were used to compare the strength development rate over time in this study. The ACI Committee 209 recommends the following relationship in Equation (2) for moist-cured concrete made with normal Portland cement (ASTM Type I):

(2) $$f_{cm}\left(t\right)=\left(\frac{t}{4+0.85\;t}\right)\;f_{c}\left(28\right)$$

The CEB-FIP Models Code [35] suggests the following relationship in Equation (3) for concrete specimens cured at 20 °C:

(3) $$f_{cm}\left(t\right)=exp\left[s\left(1-\sqrt{\frac{28}{t/t_{1}}}\right)\right]\;f_{c}\left(28\right)$$

where $f_{cm}\left(t\right)$ means compressive strength at age *t* days; $f_{c}\left(28\right)$ means 28-day compressive strength; *s* = coefficient depending on the cement type, such as *s* = 0.20 for high early strength cements, *s* = 0.25 for normal hardening cements; *s* = 0.38 for slow hardening cements and $t_{1}$ = 1 day. For control mix with water to cement ratio 0.25, as shown in Figure 5a, the increase in compressive strength with age after 28 days was observed to be lesser than the ACI-209 Model and CEB-FIP model. The addition of ultrafine has shown positive improvement in strength as the curing period increases. The addition of ultrafines even at higher dosages of replacement with cement has gained compressive strength with time. However, for a water-to-cement ratio of 0.30 as shown in Figure 5b, the gain in compressive strength was almost similar to both ACI-209 and CEB-FIP Models without addition of ultrafines. The major gain in compressive strength was observed by the addition of ultrafine at later ages after 90 days even with higher dosages. Figure 5c,d at water-to-cement ratios of 0.35 and 0.40 has shown gain of strength of concrete with time. At the early age of 28 days, with an increase in ultrafines, the compressive strength of concrete decreased. The general trend of rate of gain of strength with time increased for all dosages of ultrafines, which may be attributed due to hydration of anhydrous particles of cement and very slow hydration of ultrafine quartz leading to a drastic increase in compressive strength. It can also be observed from Figure 5 that compressive strength for almost 10% percent replacement of ultrafines with cement has no negative effect on the strength properties of concrete. It may be due to packing of ultrafine grain particles in between the cement and achieving maximum packing of material at the micro-level of mixture to attained gain in compressive strength. The rate of gain of strength with all water-to-cement ratios was observed from all the dosages of ultrafines. 

**Figure 6:**
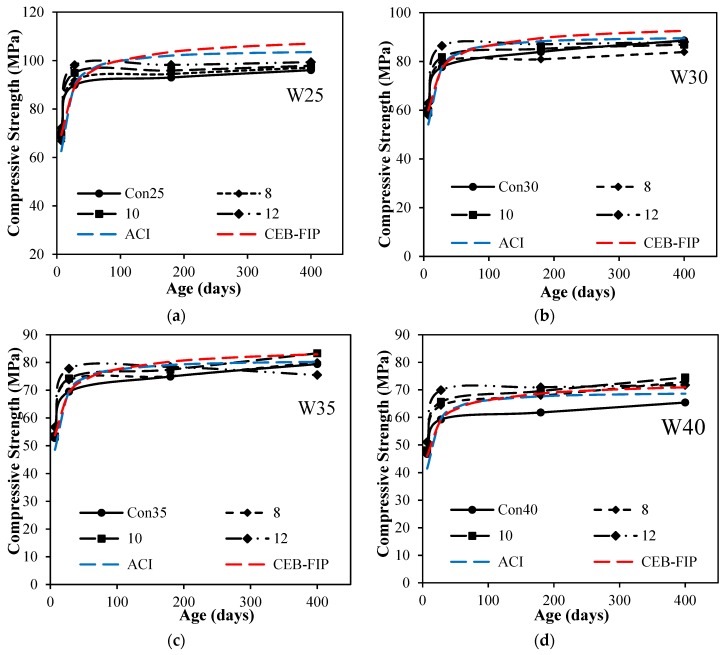
Compressive strength vs. age for different silica fume dosages with different w/c ratios: (**a**) compressive strength vs. age for different silica fume dosages at w/c 0.25; (**b**) compressive strength vs. age for different silica fume dosages at w/c 0.30; (**c**) compressive strength vs. age for different silica fume dosages at w/c 0.35; (**d**) compressive strength vs. age for different silica fume dosages at w/c 0.40.

**Figure 7:**
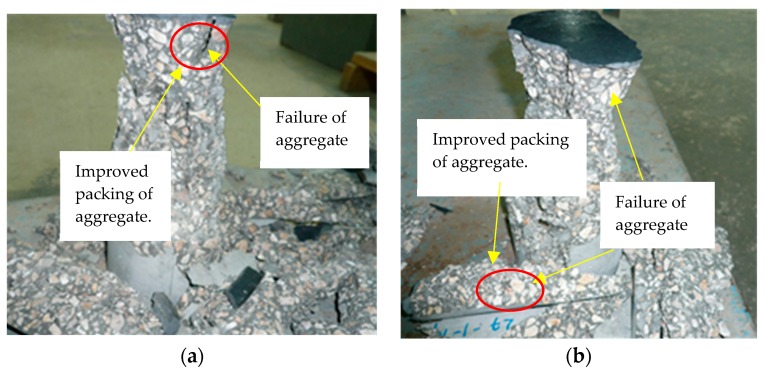
Typical failure of concrete under compression: (**a**) failure of silica fume concrete; (**b**) typical failure of concrete with ultrafines.

Figure 6 shows the results of mixes with different level of replacement of silica fume. Results revealed from Figure 6a–d that early strength of control mixtures with all the water-to-cement ratios were almost in accordance with ACI-209 and CEB-FIP models. The addition of silica fume in the concrete has significantly increased the early age strength of silica fume concrete with respect to control mixtures. Whereas, there is almost no significant increase in the strength at later ages or rather a decrease in the compressive strength, which may be due to the fact that a major hydration process has been completed within early ages of hydration. Early age increase in strength with replacement of silica fume may be due firstly to an increase in packing of particles at the micro-level, and secondly to a chemical reaction of silica fume causing an increase in bond strength between aggregate and cementitious matrix. The replacement of cement with silica fume has improved the paste structure of the matrix, which is evident from the failure pattern of concrete as shown in Figure 7. This figure shows a clear failure of aggregate in concrete prepared for all water-to-cement ratios. It is evident that at lower water-to-cement ratios, higher finer particles and improved bond strength paste with aggregate have improved compressive strength making more packed interfacial transition zones.

### 4.3. Rapid Chloride Ion Penetrability of Concrete

**Figure 8:**
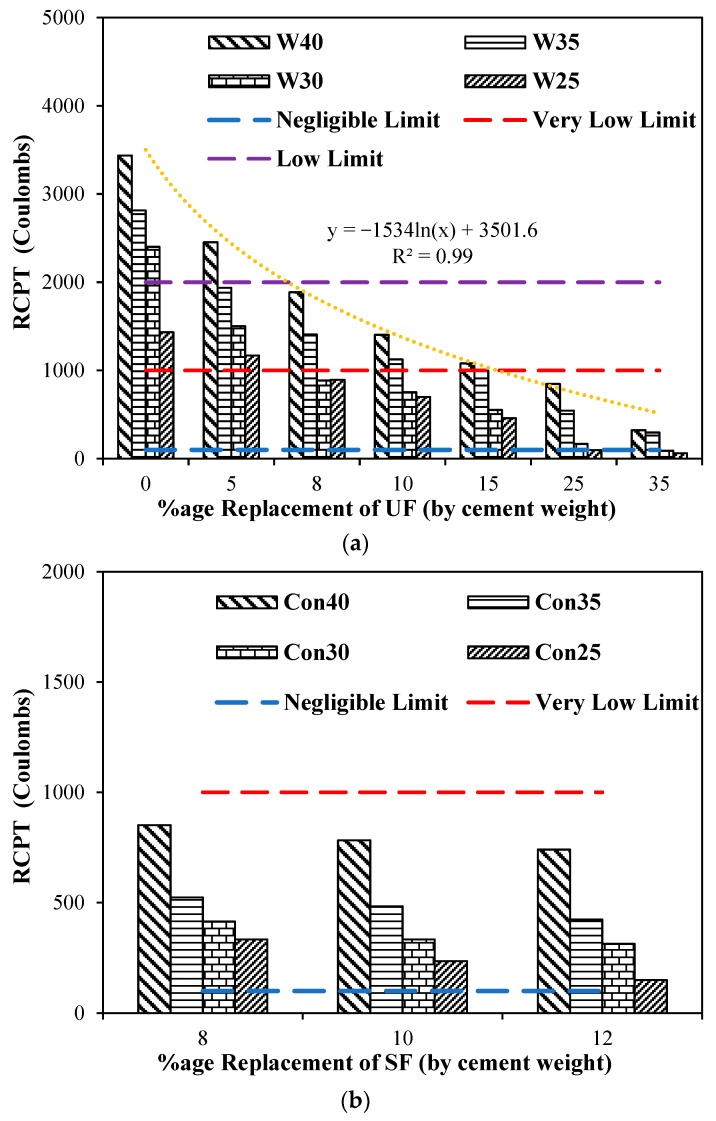
Rapid chloride ion penetrability (coulombs) for different dosages of UF and SF with different w/c ratios: (**a**) RCPT (coulombs) for different dosages ultrafines with different w/c ratios; (**b**) RCPT (coulombs) for different dosages SF with different w/c ratios.

The effect of adding ultrafine and silica fume on the chloride ion penetrability of concrete according to ASTM 1202 are presented by rapid chloride permeability test (RCPT) values for different water-to-cement ratios as shown in Figure 8. Mixtures with higher water-to-cement ratios have shown higher charge passes through the matrix. Maximum charge pass was observed through the higher water-to-cement ratio of 0.4. It is observed from Figure 8a that the increase in dosage of ultrafines has significantly reduced the passing charge through the matrix. The ASTM 1202 limits are also represented in Figure 8a to show the range of material for passing charge through the matrix. It can clearly be observed from the results that adding 8% ultrafine in the matrix for all water to cementitious ratios reduced the passing charge through the concrete to be lesser than the low limit of ASTM 1202. Also, it can be observed that for water-to-cement ratios of 0.3 and 0.25 with the addition of 8% ultrafines or more, the charge pass through concrete material is below the very low limit as per the ASTM 1202. In addition, it can be noted that using more than 15% ultrafines with all water-to-cement ratios resulted in significant reduction in RCPT below the very low limit. The reduction in the charge passes through the matrix caused by the increased dosage of ultrafines may be attributed to the fact that micro-level pores in between cement particles have been filled with ultrafines, achieving maximum packing of material at the ultrafine level. The concrete mixtures with all the water-to-cement ratios followed a general trend of logarithmic function, and replacement dosages of ultrafines is shown in Figure 8a. In general, the RCPT was influenced by addition of ultrafines, even for higher water-to-cement ratios. 

The addition of silica fume on the chloride ion penetrability of concrete according to ASTM 1202 with different water-to-cement ratios is shown in Figure 8b. Concrete mixtures with higher water-to-cement ratios have shown higher charge passes through the matrix, as can be observed for the water-to-cement ratio of 0.4. It can be observed from Figure 8b that the addition of silica fume has significantly reduced the passing charge through the matrix. The ASTM 1202 limits are also represented in Figure 8b to show the range of material for passing charge through the matrix. It can clearly be observed from the results that adding 8% silica fume in the matrix for all water-to-cementitious ratios reduced the passing charge through the concrete to be lesser than the very low limit of ASTM 1202. It can also be observed that the increase in dosage of silica fume has reduced the charge passing through matrix, which may be attributed because micro-level pores in between cement particles have been filled with silica fume, achieving maximum packing of material at the micro-level. The second reason may be attributed to the pozzolanic reaction, which improves the microstructure of concrete, making concrete denser. Many other researchers [36,37,38] who investigated the pozzolanic effect of silica fume have confirmed this improvement in the microstructure. With the increase in dosage of silica fume from 8% to 12%, there is no significant improvement in charge pass. A general trend of logarithmic function governing the behavior of all mixtures with different levels of replacements of silica fume is shown in Figure 8b. In general, the RCPT was influenced by the addition of silica fume, even for higher water-to-cement ratios.

### 4.4. Porosity

**Figure 9:**
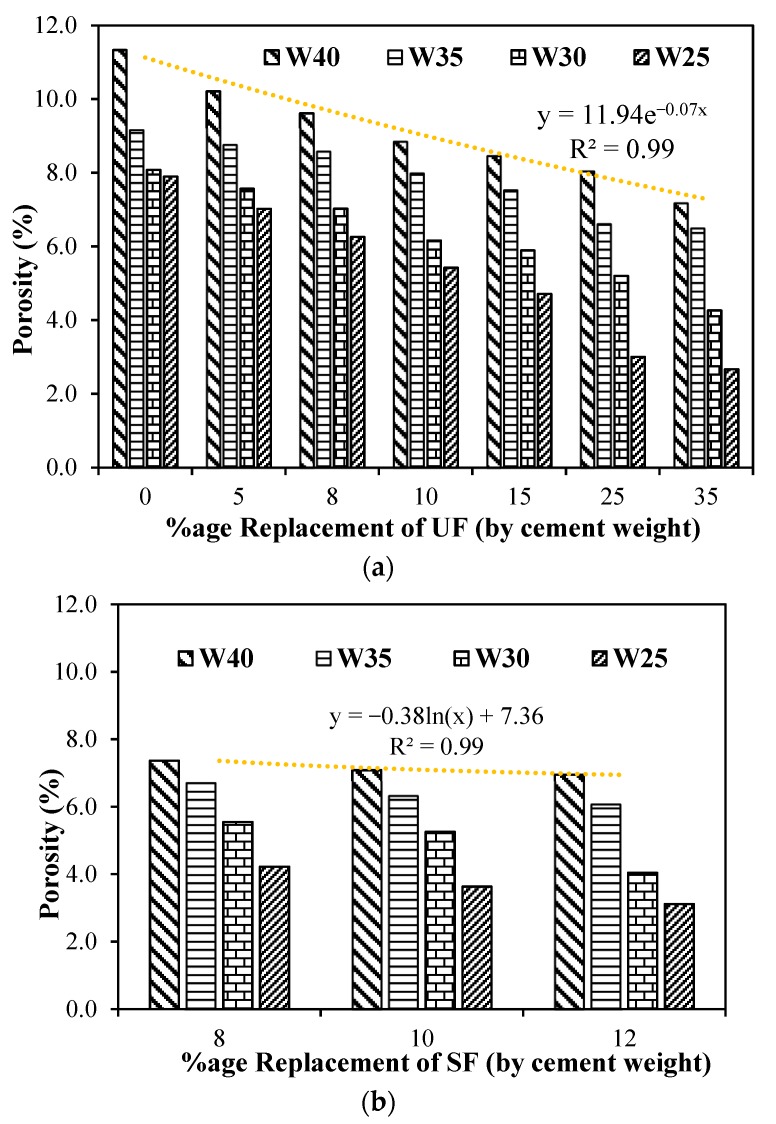
Porosity (%) for different dosages of UF and SF with different w/c ratios: (**a**) porosity (%) for different dosages of ultrafines with different w/c ratios; (**b**) porosity (%) for different dosages of SF with different w/c ratios.

The effect of adding ultrafine and silica fume in the concrete on the porosity according to RIlEM CPC 11.3 with different water-to-cement ratios are shown in Figure 9a,b, respectively. Concrete mixtures with higher water-to-cement ratios have shown higher porosity through the matrix. Maximum porosity was observed through the higher water-to-cement ratio of 0.4. It can be observed from Figure 9a,b that the increase in dosage of ultrafines and silica fume has significantly reduced the porosity of the matrix for all water-to-cement ratios. At higher water-to-cement ratios, there was lesser improvement in porosity due to the addition of ultrafines (Figure 9a) and silica fume (Figure 9b), which may be attributed due to lesser available micro-pores due to lower amounts of cement quantities in the concrete mixtures. However, better improvements in the porosity was observed for the lower water-to-cement ratios of 0.25 and 0.3; the decrease in porosity was observed to be even 50% at a replacement dosage of 25 to 35% of ultrafine in concrete. Addition of silica fume reduced porosity in the range of 40–60% for water-to-cement ratios of 0.25–0.3 and in the range of 25–40% for water-to-cement ratios of 0.35–0.4. Such improvement can be attributed due to the fact that ultrafines are filling the micro-pores resulting in increasing the density of paste at the micro-level. This reduction of porosity becomes more significant at higher cement content or high paste volume mixtures with lower water-to-cement ratios that can reach 60%, whereas at higher water-to-cement ratios of 0.35 and 0.4, maximum reduction in porosity is observed to be around 35%, as shown in Figure 9a. A general trend of exponential function showing the reduction in porosity for all concrete mixtures and replacement dosages of ultrafines and silica fume are shown in Figure 9a,b, respectively. By the comparison of Figure 9a,b, it can clearly be observed that 8% silica fume has reduced porosity by even more than 25% replacement of ultrafines, which lead to the fact that it has double effect on the microstructure. This is due firstly to improvement in packing of material at the micro-level because of finer particles and secondly to improvement of the microstructure due to pozzolanic reaction of silica fume. 

### 4.5. Relationship between RCPT and Porosity 

**Figure 10:**
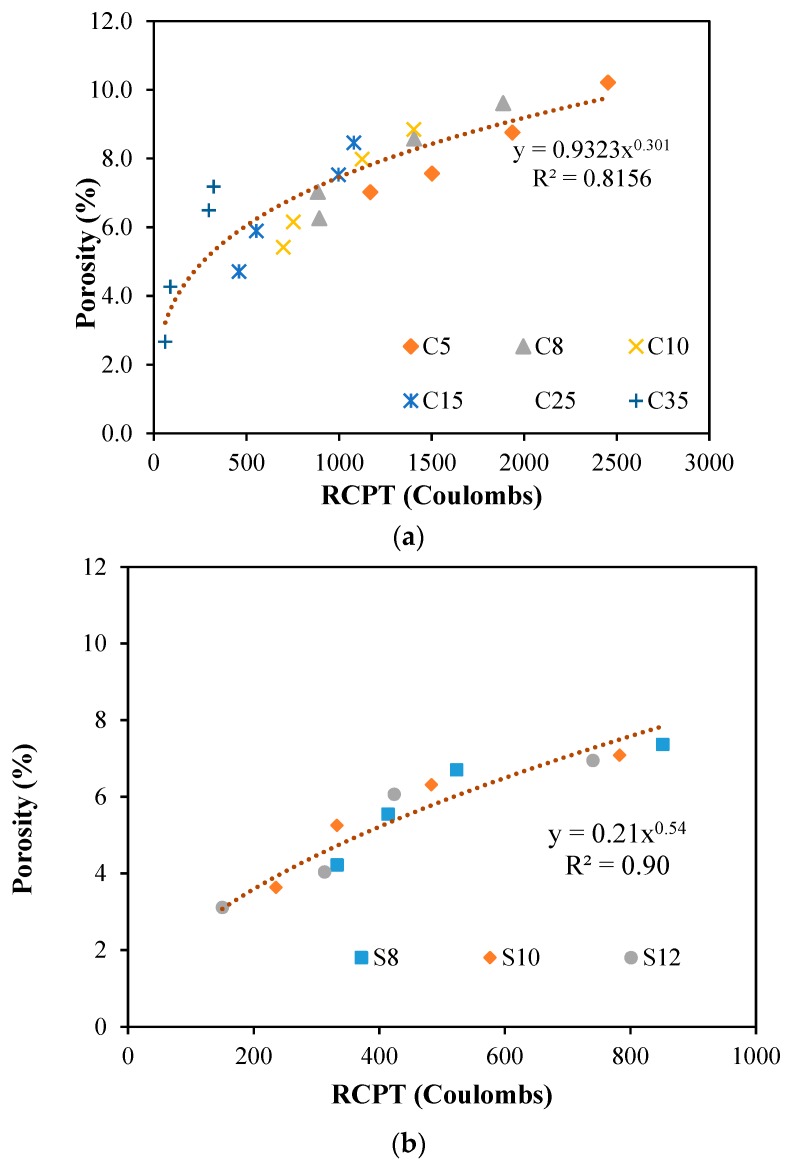
Porosity (%) vs. RCPT (Coulombs) relationship for different dosages SF and UF: (**a**) porosity (%) vs. RCPT (Coulombs) relationship for different dosages UF; (**b**) porosity (%) vs. RCPT (Coulombs) relationship for different dosages SF.

There is a link between RCPT and porosity of concrete. The porosity is the total pores available in the concrete whereas RCPT is the total charge pass through the concrete mixture. Figure 10 shows the relationship between RCPT and porosity of concrete with different water-to-cement ratios and different dosages of replacements of ultrafines and silica fume. It is worth noting that higher dosages of replacement of ultrafines has shown to reduce both porosity and RCPT values. A power function is a governing trend between porosity and RCPT values of all replacements of ultrafines in all water-to-cement ratios of concrete as shown in Figure 10a, with an R^2^ of more than 0.8. Figure 10b shows the relationship of different dosages of silica fume in different water-to-cement ratios with an R^2^ of more than 0.9. It shows a strong relationship between porosity and RCPT values for silica fume concrete. The data was plotted and a best-fit relationship between porosity and RCPT values was a power function. It clearly shows that an increase in porosity shows an increase in RCPT values for all replacement levels of silica fume. 

## 5. Predictive Models 

### 5.1. Predictive Model of Compressive Strength for Different Water-to-Cement Ratios

Predictive model for the compressive strength of concrete with different water-to-cement ratios was given by Abram [39], which is one of the significant advancements in concrete technology [40]. Characteristics and proportioning of materials and concrete, curing, and parameters of testing are major factors, which are affecting precise measurements of compressive strength. Abram’s model for prediction of compressive strength can be expressed as follow:

(4) $$f_{c}^{\prime }=\frac{A}{B^{x}}$$

where $f_{c}^{\prime }$ is compressive strength of concrete, *A* and *B* are empirical constants catering effects of aggregate, type of cementitious matrix, admixture, curing, testing condition, and age of concrete. Parameters A and B were given as 96.55 and 8.2 for Abram’s model [29,30]. To utilize this formula into this present study, a modification factor *w* is introduced in Equation (5) as follow:

(5) $$f_{c}^{\prime }=\frac{A}{B^{x}}$$

**Figure 11:**
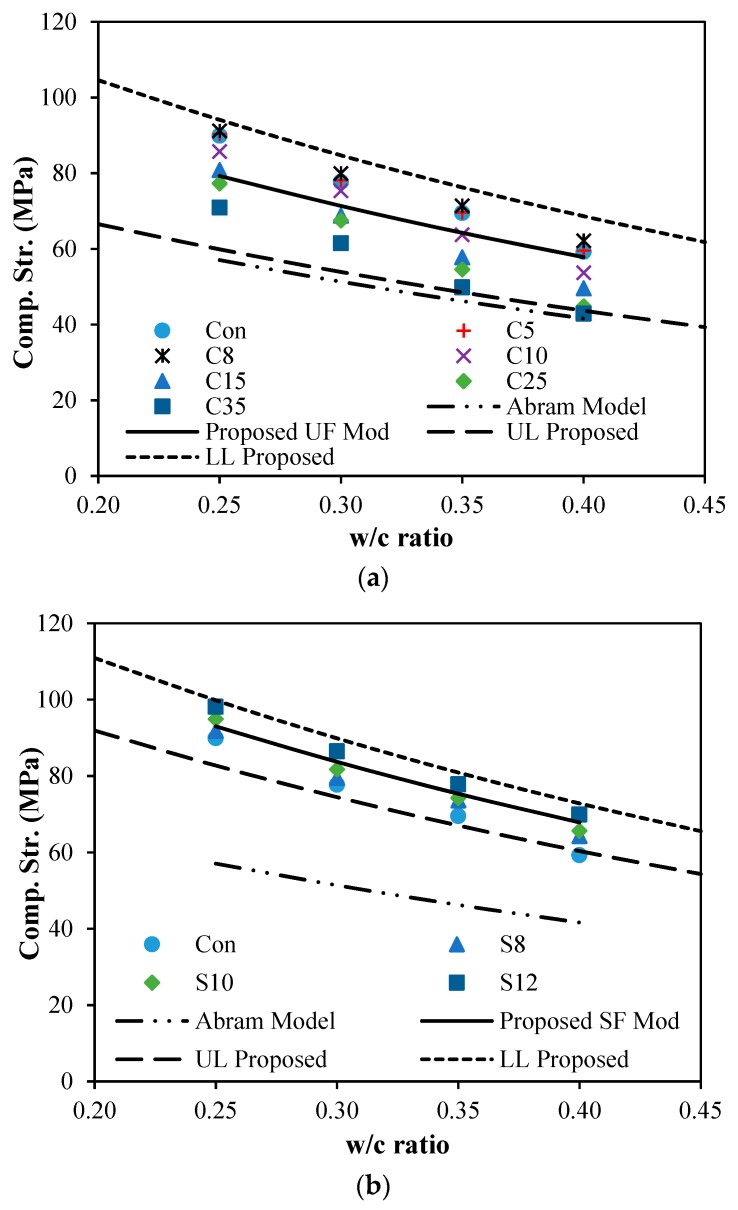
Strength vs. w/c ratio for different ultrafine dosages at 28 days: (**a**) strength vs. w/c ratio for different UF dosages at 28 days; (**b**) strength vs. w/c ratio for different SF dosages at 28 days.

A modification factor, *w*, is computed by the best-fit of experimental data of compressive strength versus the water-to-cementitious ratio for all the mixtures at the age of 28 days. Figure 5b and Figure 11a show the experimental and proposed trends with upper limit (UL) and lower limit (LL) for the silica fume and ultrafine effect on the different water-to-cement ratios. The w-value for ultrafines with different dosages in different water-to-cement ratios was 1.39. The w-value for concrete with different dosage of silica fume with different water-to-cementitious ratio was 1.63. Finally, the w-value for upper limit (UL) and lower limit (LL) for concrete with ultrafines are 1.05 and 1.65, respectively, and the upper limit (UL) and lower limit (LL) of w-value for silica fume are 1.45 and 1.75, respectively. 

### 5.2. Predictive Models for RCPT and Porosity Based on Compressive Strength 

**Figure 12:**
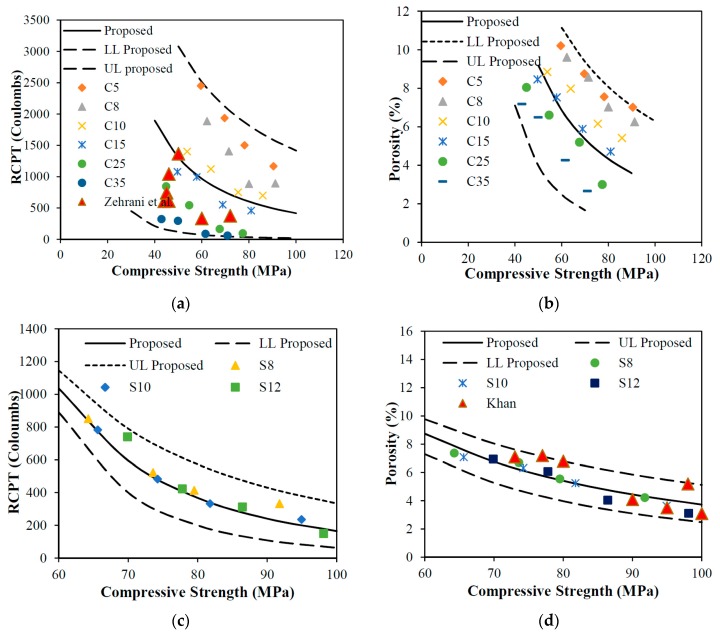
Compressive strength vs. permeation-related properties of SF and UF particle: (**a**) compressive strength vs. RCPT (Coulombs) relationship for different dosages UF; (**b**) compressive strength vs. porosity (%) relationship for different dosages UF; (**c**) compressive strength vs. RCPT (Coulombs) relationship for different dosages SF; (**d**) compressive strength vs. porosity (%) relationship for different dosages SF.

It is also very important to know the relationship between permeation-related properties and compressive strength of concrete because usually, quality of concrete and its fitness for construction is judged based on the compressive strength. So, it is important to have a relationship between compressive strength and permeation-related properties. Figure 12 shows relationships between compressive strength and permeation-related properties of ultrafines and silica fume with different replacement dosages. A predictive model has been proposed to find the compressive strength by using RCPT and porosity of concrete for different levels of replacements of ultrafines fillers as given in Equation (6).

(6) $$R,\;P=\left[w\left(\frac{\left(1+4\alpha \right)}{f_{c}^{\prime }{}^{\left(1+4\alpha \right)}}\right)\right]$$

*R* stands for RCPT and *P* for porosity value, whereas w is constant which varies for different dosages of ultrafines for RCPT and porosity of concrete. *α* is the percentage replacement of ultrafines in the concrete. The predictive proposed model is drawn in Figure 12a with the upper and lower bound values. The relationship between RCPT and compressive strength for ultrafine dosages is shown in Figure 12a. It can be observed that the developed model is in good agreement with the available literature [41], as shown in Figure 12a. The developed model has different values of *w* for RCPT and compressive strength with different replacement dosages of ultrafines with R^2^ ranging from 0.80 to 0.95. The value of *w* for RCPT varies from 250 × 10^3^ to 1150 × 10^3^ from low replacement levels of 5% to 35% of ultrafines in concrete, respectively. The relationship porosity and compressive strength for ultrafine dosages is shown in Figure 12b. The developed predictive model is the governing relationship for porosity and compressive strength with different replacement dosages of ultrafines with R^2^ ranging from 0.80 to 0.95. The value of *w* for porosity varies from 1.1 × 10^3^ to 28 × 10^3^ from low replacement levels of 5% to 35% of ultrafines in concrete, respectively. A predictive model has been proposed to find the compressive strength by using RCPT and porosity of concrete for different levels of replacements of silica fume as shown in Equation (7).

(7) $$R,\;P=\left[\frac{\left(w\right)}{f_{c}^{\prime }{}^{B\left(1+4\alpha \right)}}\right]$$

*R* and *P* stands for RCPT and porosity value, respectively, whereas *w* and *B* are constants which vary for different dosages of silica fume for RCPT and porosity of concrete. *α* is the percentage replacement of silica fume in the concrete. The proposed predictive model for compressive strength and RCPT value for different dosages of silica fume is drawn in Figure 12c with the upper and lower bound values. The relationship between RCPT and compressive strength for silica fume dosages is shown in Figure 12c. The developed model has different values of *w* for RCPT and compressive strength with different replacement dosages of ultrafines with R^2^ ranging from 0.96 to 0.98. The value of *w* for RCPT varies from 2.23 × 10^8^ to 1.47 × 10^11^ with replacement levels of 8% to 12% of silica fume in concrete, respectively. Comparison of the results with other researchers shows good agreement with developed predictive models [7], as shown in Figure 12d. The value of *B* for RCPT varies from 2.27 to 3.04 from replacement level of 8% to 12% of silica fume in concrete, respectively. The relationship porosity and compressive strength for ultrafine dosages is shown in Figure 12d. The developed predictive model is the governing relationship for porosity and compressive strength with different replacement dosages of ultrafines with R^2^ ranging from 0.93 to 0.95. The value of *w* for porosity varies from 3.7 × 10^3^ to 30 × 10^3^ with replacement levels of 8% to 12% of silica fume in concrete, respectively. The value of *B* for RCPT varies from 1.13 to 1.42 with replacement levels of 8% to 12% of silica fume in concrete, respectively.

## 6. Conclusions

The conclusions of the work can be summarized as follow:

➢ Maximum increase of 12% in compressive strength with 12% partial replacement of silica fume was observed, whereas partial replacement of cement with 35% dosage of ultrafines showed about a 28% decrease in compressive strength. This decrease in the compressive strength was attributed to a non-pozzolanic activity of inactive ultrafine filler. However, partial replacement of cement with ultrafine up to 8% showed around a 5% increase in compressive strength.
➢ All the mixtures with silica fume and ultrafines have produced higher compressive strength than given by Abram’s predictive model, and the increase in compressive strength with partial replacement of silica fume and ultrafine was 63% and 38%, respectively, as compared to Abram’s predictive equation.
➢ The partial replacement of 8% of silica fume has yielded the RCPT value to a very low limit and low porosity for all water-to-cement ratios. However, the partial replacement with 8% of ultrafines has yielded RCPT to a very low limit at a water-to-cement ratio of 0.3, but at a replacement level of 15% ultrafines. Concrete mixture with all water-to-cement ratios yielded a very low limit of RCPT value.
➢ The relationships for RCPT and porosity of all mixtures was attained which suggested that there is strong correlation between porosity and RCPT for both ultrafines and silica fume, as expected.
➢ Predictive models have been proposed for RCPT and porosity by compressive strength with consideration of dosage of replacements of ultrafines and silica fume using experimental data.

## Figures and Tables

**Table 1:** Physical and chemical properties of cementitious materials.

Oxide Composition (%)	Ordinary Portland Cement	Silica Fume (S)	Ultrafine (C)
SiO_2_	20.2	93.2	99.5
Al_2_O_3_	5.49	0.2	0.20
Fe_2_O_3_	4.12	0.03	0.03
CaO	65.43	0.72	0.01
MgO	0.71	0.14	-
Na_2_Oeq	0.26	0.07	-
SO_3_	2.61	<0.01	-
Loss on ignition (%)	1.38	5.4	-
Specific gravity	3.14	2.27	-
Fineness (m^2^/kg)	373	19,000	16,500

**Table 2:** Physical properties of fine and coarse aggregate.

Properties/Material	Specific Gravity	Absorption (%)	Unit Weight (kg/m^3^)
White Sand	2.63	0.77	1725
Crushed Sand	2.68	1.52	1552
Coarse Aggregate (10 mm)	2.65	1.45	1570

**Table 3:** Mix proportions of silica fume (SF) and ultrafine (UF) for all mixtures.

Mix	Ultrafine Content		Water to Cement Ratio							
	SF (%)	UF (%)	0.25		0.30		0.35		0.40	
			Cement Kg/m^3^	Slump (mm)	Cement Kg/m^3^	Slump (mm)	Cement Kg/m^3^	Slump (mm)	Cement Kg/m^3^	Slump (mm)
Control	0	0	550	185	500	190	450	200	400	150
S8	8	0	506	202	460	180	414	165	368	185
S10	10	0	495	175	450	170	405	200	360	160
S12	12	0	484	190	440	160	396	158	352	170
C5	0	5	522.5	171	475	184	422.5	194	380	182
C8	0	8	506	175	460	192	414	202	368	201
C10	0	10	495	198	450	179	405	191	360	184
C15	0	15	467.5	200	425	155	382.5	195	340	160
C25	0	25	412.5	200	375	180	337.5	185	300	180
C35	0	35	357.5	205	325	210	292.5	155	260	185
